# Mixing Plants from Different Origins to Restore a Declining Population: Ecological Outcomes and Local Perceptions 10 Years Later

**DOI:** 10.1371/journal.pone.0050934

**Published:** 2013-01-21

**Authors:** Anne-Claire Maurice, Jawad Abdelkrim, Matthieu Cisel, Monika Zavodna, Philippe Bardin, Alexis Matamoro, Richard Dumez, Nathalie Machon

**Affiliations:** 1 Unité Conservation des Espèces, Restauration et Suivi des Populations, Unité Mixte de Recherche 7204, Muséum National d'Histoire Naturelle, Paris, France; 2 Unité Eco-anthropologie et ethnobiologie, Unité Mixte de Recherche 7206, Muséum National d'Histoire Naturelle, Paris, France; 3 Conservatoire Botanique National du Bassin Parisien, Muséum National d'Histoire Naturelle, Paris, France; Australian Wildlife Conservancy, Australia

## Abstract

Populations of the Large-flowered Sandwort (*Arenaria grandiflora* L.) in the Fontainebleau forest (France) have declined rapidly during the last century. Despite the initiation of a protection program in 1991, less than twenty individuals remained by the late 1990s. The low fitness of these last plants, which is likely associated with genetic disorders and inbreeding depression, highlighted the need for the introduction of non-local genetic material to increase genetic diversity and thus restore Fontainebleau populations. Consequently, *A. grandiflora* was introduced at three distant sites in the Fontainebleau forest in 1999. Each of these populations was composed of an identical mix of individuals of both local and non-local origin that were obtained through *in vitro* multiplication. After establishment, the population status (number of individuals, diameter of the plants, and number of flowers) of the introduced populations was monitored. At present, two populations (one of which is much larger than the other) persist, while the third one became extinct in 2004. Analyses of the ecological parameters of the introduction sites indicated that differences in soil pH and moisture might have contributed to the differences in population dynamics. This introduction plan and its outcome attracted interest of local community, with those who supported the plan and regarded its 10-year result as a biological success (i.e., persistent populations were created), but also those who expressed reservations or disapproval of the plan and its outcome. To understand this controversy, a sociological study involving 27 semi-structured interviews was carried out. From these interviews emerged three areas of controversy: alteration of the identity of the plant, alteration of the identity of its territory, and the biological and ethical consequences of the techniques used for the experimental conservation.

## Introduction

The restoration of populations of taxa on the verge of extinction is an increasingly common practice in conservation biology [1],[2],[3]. The goal of restoration is to recover declining populations and to restore their evolutionary potential in a long-term, self-sustaining manner [4],[5],[6],[7]. This requires the selection of sites with ecological characteristics corresponding to the needs of the species [4],[8],[7],[9]. Restoration plans usually involve the restoration of the regional viability of a species through an increase in population size and genetic diversity [10],[11]. For example, a sufficient number of individuals is required to avoid effects of demographic stochasticity and Allee effects [12],[13],[14]. Furthermore, increased genetic diversity may enhance the viability of a population facing environmental changes and counteract inbreeding depression that could have arisen from mating among relatives [15],[16],[17],[2],[18]. Thus, restored populations could benefit from the relocation and introduction of individuals from different populations [19],[3], and their genetic composition could be improved through heterosis [20]. However, this practice may be beneficial only if recombination among genomes actually leads to increased fitness of the progenies (heterosis) rather than reduced fitness caused by outbreeding depression [6],[21]. Unfortunately, the occurrence of outbreeding depression is relatively unpredictable, even if its probability is small in crosses between populations that separated in the last 500 years, are located in similar environments and between populations suffering from inbreeding depression [22],[23].

Consequently, conducting a restoration plan for a locally disappearing species might require the transfer of individuals from one site to another for one of the following reasons: (1) to restore the population of plants at a site that is becoming or has already become unsuitable; or (2) to increase the size of the gene pool and avoid inbreeding in a declining population.

Such new conservation biology approaches, which are highly interventionist, are contrary to the historical approach to conservation, which arose from the conflicts between humans and nature and assigned to naturalists a clear ethical position: defending nature against human influence. On the contrary, by suggesting proactive management plans (e.g., the introduction of species), they introduce new ethical questions [24],[25].

Several social scientists have studied controversies about the introduction of animals, particularly those involving large carnivores [26],[27],[28],[29],[30],[31]. However, plant restoration programs and resulting controversies have been documented less often, and to our knowledge, no studies have explicitly integrated analyses of the social interactions surrounding these controversies.

**Figure 1:**
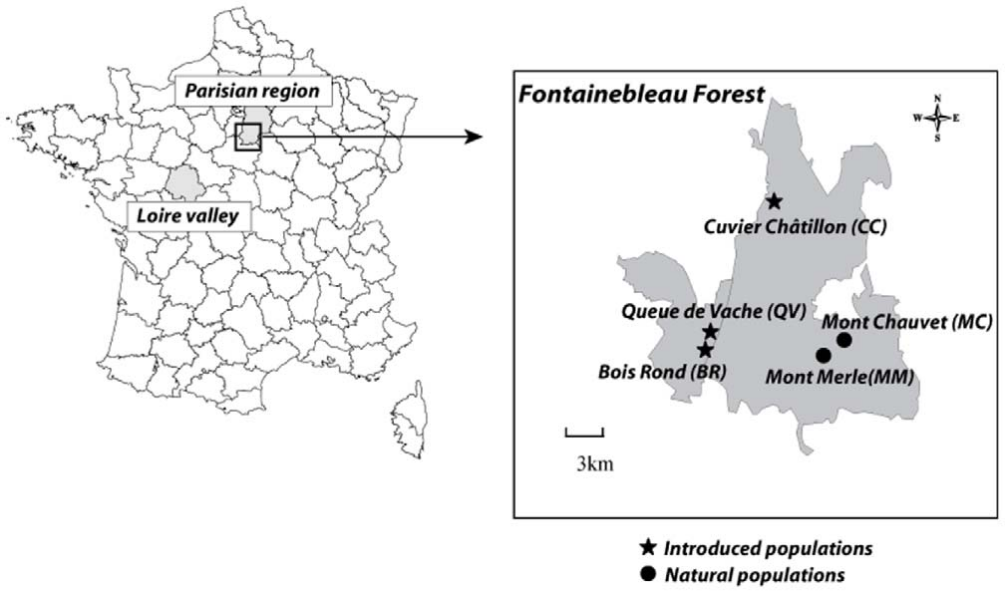
Location of *Arenaria grandiflora* L. in the Fontainebleau Forest (France).

Our study focuses on the Large-flowered Sandwort (*Arenaria grandiflora* L.), a perennial Caryophyllaceae on the verge of extinction in the Parisian region (France). These populations of *A. grandiflora* have declined so rapidly in the last decades of the 20^th^ century that in 2009, only one remnant individual was observed in the last native population situated in the Fontainebleau forest, 50 km south of Paris (Fig. 1). Previous studies based on common garden experiments indicated that crosses within populations of the Fontainebleau forest had reduced seed set and seed mass compared with crosses between plants from Fontainebleau and other French populations [10]. Such observations were attributed to increased inbreeding or fixation of deleterious alleles by drift following the severe decline of the Fontainebleau populations. This hypothesis was supported by an analysis of isozymes, which revealed very low genetic diversity in the Fontainebleau population compared to other French populations (Machon, unpublished results).

As a result, a restoration plan was executed by conservation biologists, botanists of the National Botanical Conservatory (CBN), and managers of the Fontainebleau forest with two objectives: (1) to prevent *A. grandiflora* from disappearing from the Fontainebleau forest and (2) to test and evaluate a restoration plan that involved introducing a mix of plants from different locations. This experimental introduction was conducted at three sites, which appeared ecologically similar to the natural sites. About 440 plants from two origins (2/3 local and 1/3 non-local), were introduced to each site. The three created populations and their status were monitored for ten years following their introduction in 1999 to (1) record their dynamics and (2) evaluate the reproductive success (measured by flowering rates) and life span of the local and non-local plants and their progeny.

As this program was being developed, disapproval and criticism arose from members of the local community concerned with the conservation of *A. grandiflora*. The experimental introduction was more or less supported by the different social participants [32] but was sometimes regarded as inappropriate. However, the origins of the disagreement were not clearly identified. To understand the different points of view, a sociological study based on qualitative methods was undertaken.

In this paper, we report on the biological outcome of the restoration of *A. grandiflora* ten years after the introduction with an analysis of the different positions and opinions of the various participants that were involved in the conservation of this plant species. To evaluate the success of this restoration plan, we jointly (1) report on a ten-year monitoring of *A. grandiflora* in the Fontainebleau forest and (2) explore the controversies that arose from the project. This case study aims to assist conservation professionals in addressing biological as well as sociological questions concerning plant conservation.

## Methods

### Study species

*Arenaria grandiflora* L. (Caryophyllaceae) is a summer-blooming perennial that is native to cliffs and calcareous rocks of the southern and central mountains of Europe. In France, it also appears (albeit rarely) in the lowlands at two extant locations (Fig. 1). One is in the Parisian region (Fontainebleau forest), and the other is 200 km southwest of Paris, in Chinon (Loire valley), and comprises over one hundred individuals. These lowland populations have been legally protected since 1991. The first recorded observations of the Parisian *A. grandiflora* date from the end of the 17^th^ century [33]. It was described by Sébastien Vaillant in 1727 and has been regularly mentioned in botanical publications since that time. According to a recent botanical report, the population at the Mont Chauvet site comprised tens of individuals circa 1950. In 1995, two small populations were recorded in the Fontainebleau forest: Mont Chauvet and Mont Merle, comprising only 20 and 18 individuals, respectively. In 2003, despite having built enclosures around the last individuals to protect them from herbivores and walkers, the Mont Chauvet population became extinct. By 2009, the Mont Merle population had been reduced to a single, stunted plant.

The first alert concerning the decline of *A. grandiflora* in the Fontainebleau forest was given in the 1990s by a local naturalist (designated here as a person who has very good knowledge of nature, gained in part by regular direct observation) who informed forest managers at the French National Forest Office (ONF) of his concerns about the risk of the disappearance of the species. After an unsuccessful attempt to rescue this heliophilic plant by clearing the habitat to provide more sunlight, the ONF relayed the alert to the botanical conservatory. As a result, an enclosure was built to protect the last remaining individuals, and a population geneticist from the botanical conservatory was asked to develop a restoration program.

### Experimental design of the introduced populations

All necessary permits were obtained for the restoration program. The proposed plan was submitted with success in 1999 to the CNPN (Conseil National de Protection de la Nature), the French consultative committee in charge of assessing experimental projects on protected species for the French ministry responsible for overseeing environmental issues. It was then approved by the botanical conservatory responsible for its implementation and by the French National Forest Office (ONF), the forest managers responsible for the experimental sites.

Thus, the establishment of new populations of *A. grandiflora* began in 1997 when cuttings were taken from wild plants in the two known lowland localities: Chinon in the Loire valley (n = 11 plant individuals) and the declining populations from the Fontainebleau forest (n = 9 plant individuals). For two years, these plants were multiplied through *in vitro* culture, yielding a total of 1320 individuals. In 1999, the plants were introduced into the Fontainebleau forest at three sites that were considered ecologically suitable for *A. grandiflora*. The sites – “Bois Rond” (BR), “Cuvier Chatillon” (CC), and “Queue de Vache” (QV) – are located 10 km distant from the extant native populations (Fig. 1). The QV site is located 6 km from CC and 0.6 km from BR (Fig 1). At each site, an identical population (i.e., the same proportion of clones from each origin obtained through *in vitro* culture) of 220 plants was transplanted in two 100 m^2^ enclosures (i.e., a total of 440 plants per site). Each of these six replicates was composed of 1/3 non-local plants (i.e., originating from the Loire valley) and 2/3 local plants (i.e., from the Parisian region). In this study, the two enclosures located at the same site were considered as a single population because of their proximity (a few meters in CC and several dozen meters in QV). The plants from both origins were randomly mixed and planted in patches of bare sandy soil, with a minimum spacing of 20 cm.

### Environmental comparison among sites

To assess differences among the three experimental sites, soil pH and moisture were measured in 2005 and 2009, respectively.

To measure pH, eight samples of soil (four samples from each of the two first soil horizons) per site were collected following standard protocols [34]. The soil samples were dried at ambient temperature and sifted. A magnetic agitator was used to mix 20 g of dried soil with 50 g of distilled water for 5 min before measuring the pH (pHeI, Hanna Instruments). Because no significant differences were observed between top and deep soil, these data were pooled. The data showed a non-normal distribution, and differences among sites were tested with a Kruskal-Wallis rank test.

To measure soil moisture, four samples were collected per site. Soil moisture was determined using the gravimetric method [35], based on the loss of mass from 20 g of fresh soil after 48 h at 105°C. Differences between sites were again tested with a Kruskal-Wallis rank test.

### Dynamics of the restored populations

Each year between 1999 and 2009, the survival state (alive or missing) of each founder individual of *A. grandiflora* was recorded at each site. Plant diameter and number of flowers, which is a good predictor of seed set (correlation calculated in 2000 on ¼ of the fruit set of 500 plants, a = 1.18, R^2^ = 0.53, (Machon, unpublished result)), were recorded in May and July, respectively. Furthermore, each year, new plantlets were identified, labeled, and monitored.

To estimate the performance of the individuals introduced in 1999, plant diameter (n = 421, 425, and 433 plants for BR, CC, and QV, respectively) and number of flowers (n = 222, 220, and 215 plants for BR, CC, and QV, respectively, on plants from one of the two enclosures randomly chosen at each site) were recorded in 2000. The effects of site (BR, CC, and QV), geographical origin (Fontainebleau and Chinon), and their interaction were tested with a two-way ANOVA after log-transformation to obtain normality of the residuals [36].

In 2009, the state of the populations at the introduction sites was assessed by recording the plant diameter, flower number, and life span of the individuals. With the exception of the remaining founders (n = 12), the origin of the individuals was unknown because they were the offspring of uncontrolled crosses. Therefore, only the effects of site (and not origin) on plant diameter (n = 91 and 968 for CC and QV, respectively) and number of flowers (n = 91 and 972 for CC and QV, respectively) were tested using the *t*-test, after square-root transformation. Although the variance in plant diameter met the variance equality assumption (F_86,964_ = 1.29, *p* = 0.085), the variance in number of flowers did not (F_88,966_ = 2,67, *p*<0.001). Therefore, the Welch *t*-test variant was used for the latter variable. Note that the population at the BR site became extinct in 2004, and therefore was not included in these statistical tests. To compare founders' lifespans between sites, the death-age of each *A. grandiflora* planted in 1999 was calculated (n = 1299) as the number of years between the first and last time a plant was detected. Thus, an age of 10 was attributed to the founding individuals still alive in 2009. The effects of site, origin, and their interaction on the founders' lifespan were tested with a two-way ANOVA after square-root transformation to obtain normality of the residuals.

### Local attitudes toward the experimentation

A study was performed in the spring of 2008 and 2009, during two periods of two months each, with the agreement of all experimental partners (the botanical conservatory responsible for the implementation of the restoration and the French National Forest Office (ONF), which was responsible for site management). The study was based on an analysis of 27 semi-structured interviews of 23 people involved in or concerned about the conservation of *A. grandiflora* in the Fontainebleau forest. The interviews were conducted according to ethical principles, i.e., the interviewees agreed verbally (1) to participate in the study, (2) to have their interviews recorded and quotations used for research and in scientific publications, (3) that their anonymity had been respected during all stages of the study. The records represented approximately 35 hours of interviews. Five people identified as having a key role in the conservation of *A. grandiflora* or as providing new data regarding social representation were interviewed twice. We interviewed 6 forest managers (7 interviews) from the French National Forest Office (ONF), the main institution managing the Fontainebleau forest; 4 professional botanists (5 interviews) from the National Botanical Conservatory of the Parisian Basin (CBNBP), an organization accredited by the government undertaking *ex situ* and *in situ* regional conservation plans of protected plant species; 2 researchers (2 interviews), who specialized in conservation biology and/or population genetics; and 11 individuals from local associations (13 interviews) that work for the protection of the natural and cultural heritage of the “Massif de Fontainebleau” (Fontainebleau area). These latter individuals have very good knowledge, acquired both professionally and privately, of the taxonomic group that they are passionate about.

Semi-structured interviews consist of a list of topics and questions prepared by the interviewer. The order of the topics can be modified during the interview, and new questions can be integrated, following the discourse of the interviewee, who can spontaneously address new topics. However, the predetermined topics must be integrated during the course of the interview. This approach, which aims at studying social processes and the logic of representation, follows the “rigor” of qualitative collection and analysis of data [37]: 1) the survey is considered complete when the researcher does not add any information about new worldviews or practices (the concept of “saturation” of the sample); and 2) moreover, in the particular case of *A. grandiflora*, the survey covers the entirety of the influential protagonists and people that are aware of the existence of the plant.

The results were transcribed as a synthesis, combining the results and their analysis, as is usually performed when these methods are employed (qualitative study with a limited number of samples). Here, we did not analyze all of the various representations of the people interviewed but focused on those expressing opposition to the experimental introduction and the corresponding divergent opinions expressed by the supporters of introduction.

## Results

### Dynamics of the restored populations

**Figure 2:**
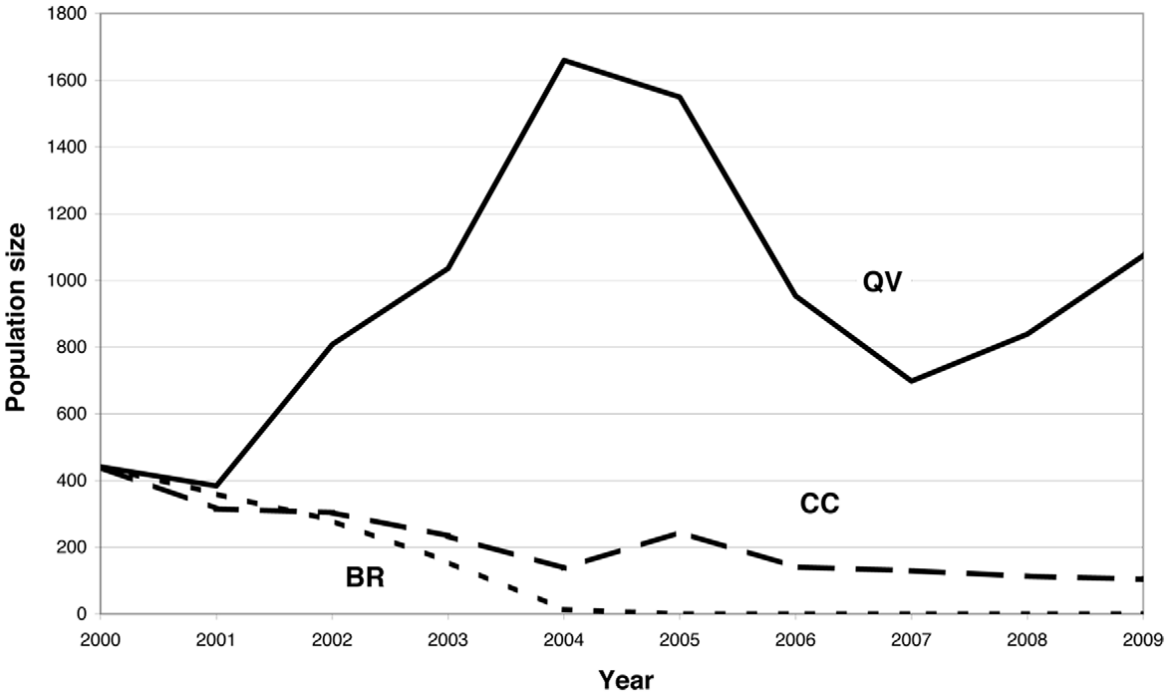
Population size of *A. grandiflora* over time since the creation of populations in the Fontainebleau forest. BR: Bois Rond population; CC: Cuvier Chatillon population; QV: Queue de Vache population.

The 10-year dynamics of the three newly introduced populations with identical initial genetic composition are shown in Fig 2. The BR population became extinct after five years. The CC population gradually declined, with 92 plants remaining in 2009, and the QV population fluctuated in size but always remained larger (approximately 1000 individuals in 2009) than the initial population founded in 1999.

### Environmental comparisons among sites

**Figure 3:**
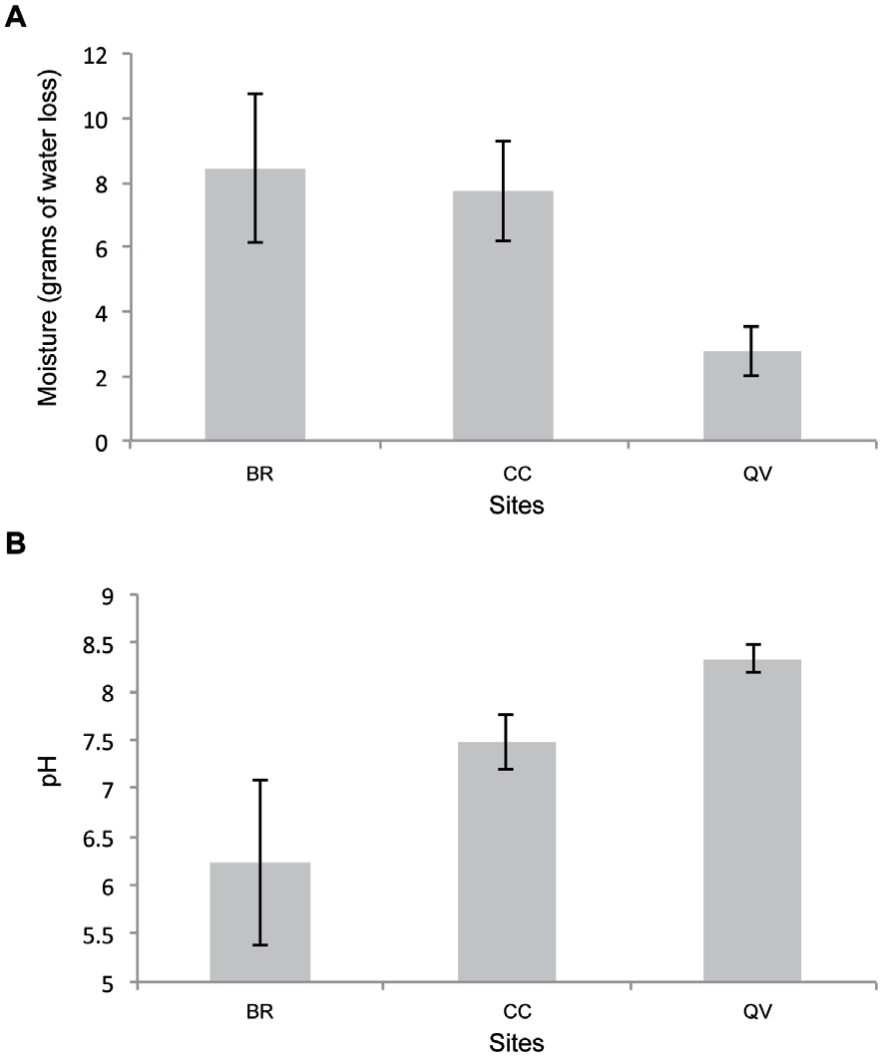
Barplots showing (**A**) moisture in grams of water loss per 20 grams of fresh soil after 48 hours at 105°C and (**B**) pH of the soil recorded at the three introduction sites (BR: Bois Rond, CC: Cuvier Chatillon and QV: Queue de Vache). The bars represent the means, and the whiskers represent 95% confidence intervals.

The three introduction sites displayed strong environmental differences (Fig. 3A and B). QV was much drier than the other sites (χ^2^ = 8.8, df = 2, *p*<0.05), and the pH of its soil was more basic than that of the other sites (χ^2^ = 19.16, df = 2, *p*<0.001).

### *A. grandiflora*: performance of introduced individuals

**Figure 4:**
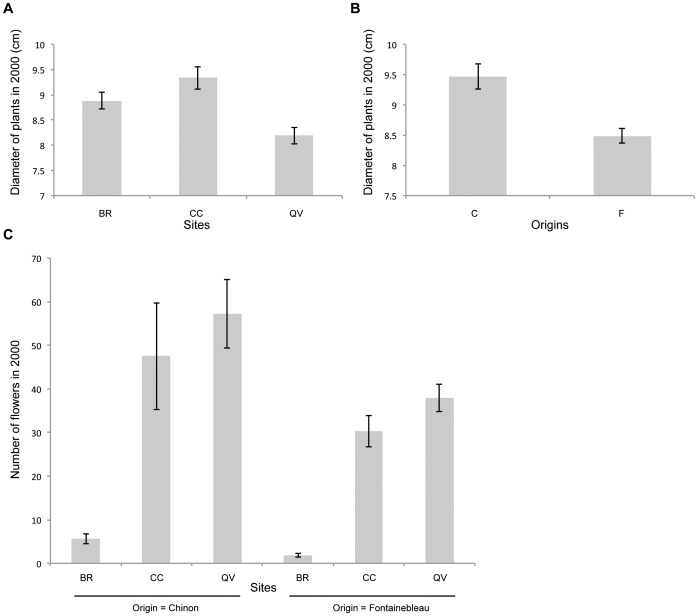
Barplots showing the effect of (**A**) different sites (BR: Bois Rond, CC: Cuvier Chatillon, QV: Queue de Vache) and (**B**) origins (C: Chinon, i.e., non-local; F: Fontainebleau, i.e., local origin) on plant diameter (cm) in 2000; (**C**) number of flowers in 2000. The bars represent the means, and the whiskers represent 95% confidence intervals.

In 2000, both the origin of the plants and the site had significant effects on plant diameter (F_2,1286_ = 40.11, *p*<0.001 and F_1,1286_ = 74.93, *p*<0.001, respectively). However, no effect of interaction between site and origin was found (F_2,1286_ = 2.36, *p* = 0.094). The plants at QV were smaller than those at the other sites (Fig. 4A). Plants from Fontainebleau (local origin) were smaller than plants from Chinon (non-local) (Fig. 4B). The number of flowers per plant was also affected by site and origin (F_2,650_ = 689.74, *p*<0.001 and F_1,650_ = 56.51, *p*<0.001, respectively). Local plants produced fewer flowers than non-local plants (Fig. 4C). Plants of both origins produced more flowers at the QV site than at the other sites, and plants at the BR site produced the fewest flowers per plant; the effect of origin/site interaction was significant (F_2,650_ = 8.45, *p*<0.001) because differences between sites were larger for non-local plants than for local plants.

### *A. grandiflora*: status of the various populations in 2009

**Figure 5:**
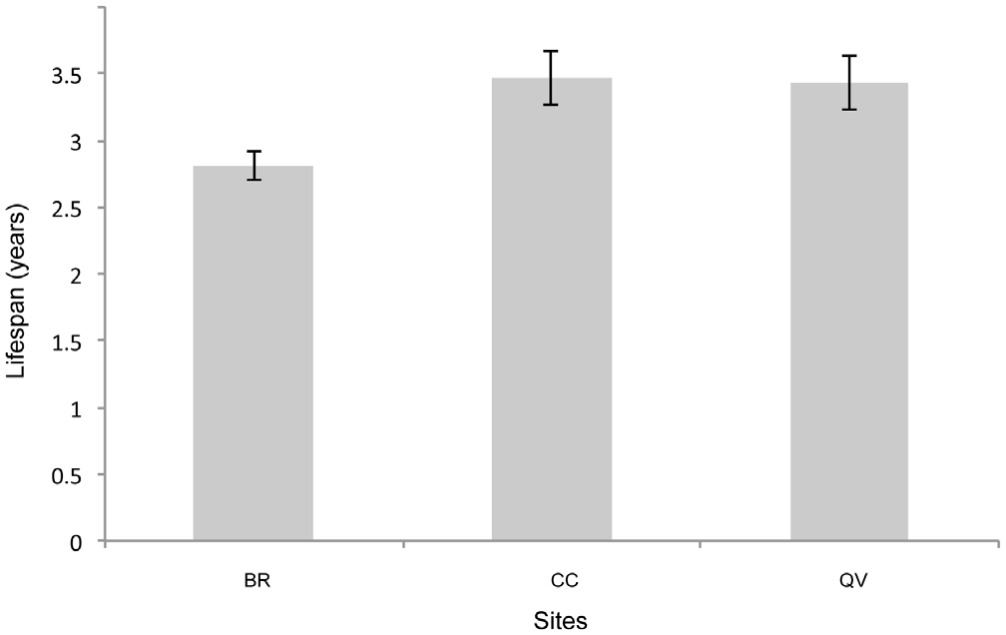
Lifespan (in years) calculated in 2009. The bars represent the means, and the whiskers represent 95% confidence intervals. For significance, see the main text.

No significant difference in life span was observed between plants of local and non-local origin (F_1,1293_ = 2.59, *p* = 0.11). Plant lifespan (survival) differed significantly among the sites (F_2,1293_ = 9.14, *p*<0.001). However, when the BR site (extinct in 2004) was not included, the mean lifespan of the plants did not differ between the two remaining sites (F_2,1293_ = 0.0002, *p* = 0.99); (Fig. 5).

**Figure 6:**
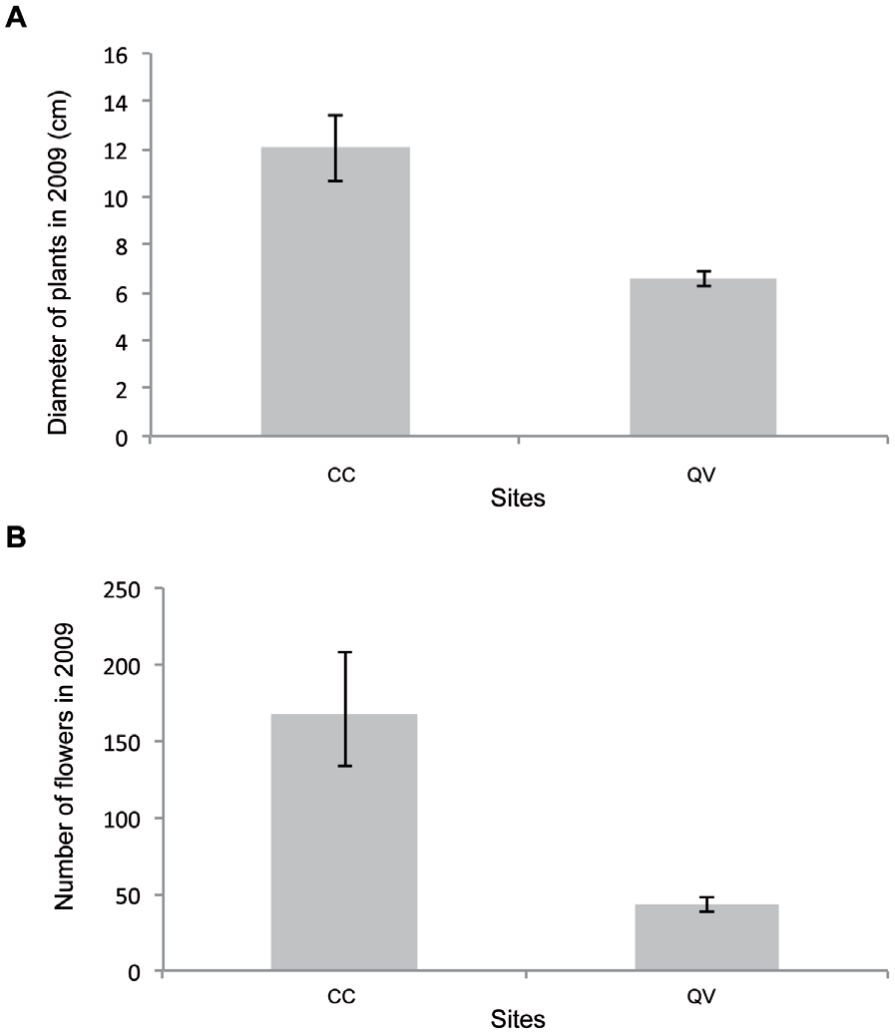
Barplots showing the effect of different restoration sites (CC: Cuvier Chatillon and QV: Queue de Vache) in 2009 on (**A**) plant diameter (cm); (**B**) number of flowers. The bars represent the means, and the whiskers represent 95% confidence intervals.

In 2009 (Fig. 6 A and B), similar to 2000, plants from QV were significantly smaller than those from CC (t_1050_ = 8.982, *p*<0.001). However, in contrast to what was observed in 2000, plants at the CC site produced more flowers than plants at the QV site (t_94_ = 8,078, *p*<0.001).

### Sociological approach

The interviews confirmed that scientists, botanists, and forest managers involved in the introduction project see the restoration plan as successful. The previous attempts by ONF to manage the ecological characteristics of the natural sites (mainly cutting of trees and building of enclosures against herbivores) did not halt the decline of the species, and therefore the genetic composition of the populations (i.e., inbreeding depression) was the first problem to be addressed by the current plan. Conversely, with some exceptions, few local naturalists shared the opinion that the disappearance of *A. grandiflora* was mostly due to ecological causes and that better management of the natural sites was the key to restoration. The genetic dimension of the restoration plan was considered as being too distant from the question of the ecological needs of the species.

## Discussion

### Ecological considerations

At each of the three sites at which *A. grandiflora* was introduced in the Fontainebleau forest, differences in the 10-year population status were observed, ranging from extinction within 5 years after introduction to a more than doubling of population size. Thus, in 2009, the total number of plants at all persisting sites was approximately the same as in 1999, when the introduced populations were founded. Therefore, overall, the introduction experiment has been successful in preventing the immediate disappearance of *A. grandiflora* from the Fontainebleau forest and perhaps in restoring its long-term potential.

The introduction of these plants at multiple sites and the establishment of long-term monitoring aid the identification of key factors that may affect the success of population restoration and should therefore be considered in future plant introduction programs. The choice of restoration site appears to be a factor that can lead to the failure of the introduction. Given the difficulties of manipulating plants of a rare and protected species, only three experimental populations were created, without replication of similar sites; efforts were made to reduce confounding effects by performing the transplantation on a single day, using the same team of gardeners, in the same type of forest area exclosures. The results indicate that site quality can strongly influence the population dynamics of *A. grandiflora*. Indeed, although the introduced populations were initially of genetically identical composition, their dynamics differed markedly. The environmental factors assessed in this study (pH and soil moisture) may have affected the viability of the populations of this species, which is naturally distributed primarily on cliffs and calcareous rocks of mountains.

In particular, the selection of a site with a soil that was too moist and acidic (BR) may have contributed to the observed rapid local extinction. The population failed to expand, likely because environmental conditions were unsuitable for *A. grandiflora* reproduction. Indeed, in the first year after introduction, plants at BR produced significantly fewer flowers, while plant diameter was similar to that of plants at the other sites. Therefore, although the ecological conditions at BR did not seem to affect the lifespan of the founders, the population died out with its founders because they never produced offspring.

At the CC site, environmental stochasticity appeared to play a significant role in population dynamics because the population size declined after introduction but remained viable, with approximately 100 individuals. The founders produced seeds. We suspect that a low seed germination rate and/or low survival of the plantlets may explain the current small size of this population.

Finally, the QV site was the only one at which the population of *A. grandiflora* has expanded since its introduction and remained large over the 10-year period. Although the plants are smaller, they reproduce in abundant cohorts.

This study also suggests that introducing individuals from different locations may improve the overall outcome of plant introductions. One year after introduction, the non-local introduced plants were larger than the local ones and produced more flowers. However, overall, the founders had a similar lifespan, regardless of origin. Newly developed microsatellite markers will enable the evaluation of the genetic composition of the populations over the 10-year period and the identification of offspring (pure non-local, pure local or admixed) [38]. Nevertheless, the increase in population size at QV indicates that mixing local and non-local individuals does not seem to cause outbreeding depression in *A. grandiflora*. In 2009, the majority of the individuals seemed vigorous and exhibited a large number of flowers. According to the predictions of other authors [22],[23],[39], because the populations used for the experiment were of ecologically similar origin and the supplemented population was potentially suffering from inbreeding depression, admixture could have occurred.

### Sociological considerations

As presented in the results, the interviews confirmed that scientists, botanists, and forest managers considered the restoration plan successful. However, a few local naturalists share the opinion that the conservation practice had been a disaster for *A. grandiflora* of the Fontainebleau forest.

#### Alteration of the plant

The first point of divergence appears to be the introduction of plants from another region. Although a majority of the naturalists would have accepted mixing plants from different populations from the Fontainebleau forest, the introduction of plants from an external site (Chinon) was seen as unsuitable because the naturalists considered this a modification of the object of interest. This idea is exemplified in the words of a naturalist who qualified the introduction of Chinon individuals as “hybridogenous”.

This last adjective is usually used to designate a taxon resulting from the cross between two different parental taxa. Such a phenomenon is described as a form of extinction of the species [40]. Its use to characterize the *A. grandiflora* experiments implies that hybridization occurred and that the cross of *A. grandiflora* from Fontainebleau with plants from Chinon would result in the disappearance of *A. grandiflora* from Fontainebleau. In other words, this naturalist insinuated that *A. grandiflora* from Fontainebleau is a distinct taxon.

Some of the local individuals that were interviewed argued that, owing to particular morphological or ecological characteristics observed in nature (e.g., specificity of the pollinator) and in a local herbarium, *Arenaria grandiflora* from Fontainebleau is endemic. However, they also categorized the Fontainebleau's natural population as remarkable because of other cultural features. The species mostly grows in the southern and central mountains of Europe, and Fontainebleau is one of the very rare plains locations, therefore constituting a botanical curiosity. Furthermore, it is considered a historical population because famous botanists such as Joseph Pitton de Tournefort (end of the 17^th^ century), Sébastien Vaillant in 1723 [41], and Carl Von Linné in 1771, observed, collected, and described this species at this locality. These two aspects make *A. grandiflora* from Fontainebleau a “patrimonial” plant. Thus, some of the reluctance concerning the introduction experiment stems from the wish to conserve a symbolic plant, part of the local botanical and natural history, rather than the species *A. grandiflora* itself. According to the sociologist [42], the categorization of species as emblematic or “patrimonial” is a social construction generally unrelated to the natural interest of the presence of a species at a site. However, because no one has truly demonstrated that *A. grandiflora* has a specific role in this ecosystem, this consideration could as well be applied to the motivations of the advocates of the introduction.

For the populations genetics researchers interviewed, the fear of hybridization that is common when preserving local taxa is based on the fear that hybridization of exotic plants with local plants could annihilate local adaptations or break down co-adapted gene complexes. However, two conclusions from *in vitro* experiments convinced the first researcher involved in the project to consider mixing the populations: 1) isozyme analysis shown low levels of genetic diversity in the Fontainebleau population, most likely due to its long isolation; and 2) crosses and cultivation in controlled conditions demonstrated that individuals from the Fontainebleau forest exhibited signs of inbreeding depression [10]. Furthermore, no natural immigration was expected to counteract this genetic weakness. As a result, an introduction of plants from Chinon was planned because these plants were thought to be genetically similar to those at Fontainebleau due to the strong similarity of their biotope (plains, forest environment, similar soil). This analysis and interpretation by instigators of the restoration program was thus mainly influenced by population genetics theories, in addition to ecological concepts. Moreover, the previous results of the biological experiment are now regarded by its instigators as providing evidence that mixing of populations is not deleterious to *A. grandiflora*. Consequently, they favored preservation of a higher taxonomic level than that advocated by the naturalists.

#### Alteration of the forest

Interviews with the naturalists indicated that the problem of introduction extends beyond the use of non-local individuals of *A. grandiflora* to include the introduction to the Fontainebleau forest of exotic species in general and often the discussion drifted to the introduction of Scots Pine (*Pinus sylvestris*) at the end of the xviii ^th^ century for forestry purposes.

Defenders of that introduction (mostly forest managers and one environmentalist) argued that Fontainebleau is part of the natural distribution of Scots Pine because it was present in the region during the post-glaciation period. Opponents (other naturalists) claimed that these conifers constitute a threat to the biodiversity of the forest, acidifying the soil and eliminating the understory. Scots Pine is seen as particularly noxious to *A. grandiflora* and possibly responsible for its decline. In the opponents' view, the “Massif de Fontainebleau” was historically a “patchwork” of open landscapes and dispersed forest habitats, modeled by carriers, coalmen, and traditional stock breeders. However, the large plantations of various forestry species in general, and Scots Pine during the 20^th^ century in particular, would have led to the disappearance of open habitats.

Finally, discussions of *A. grandiflora* indicated that several naturalists wished to regain the forest they knew several decades ago, when open habitats were more common. Pines have since invaded a number of these habitats, profoundly transforming the landscapes, the flora, and the fauna. *A. grandiflora*, regarded as native to Fontainebleau and adapted to open landscapes, is somewhat of a symbol of the ideal Fontainebleau forest. Thus, altering this “authentic plant” (quotation from a naturalist) means altering the Fontainebleau forest.

#### Alteration resulting from the experiment

Many naturalists find this method too interventionist and advocate other types of alteration. Concerning small isolated populations suffering from inbreeding depression, [43] questioned the relevance of the problem of introducing non-local genes when compared to the alternative of letting the populations disappear in the course of the natural fluctuations of the species distribution. The answer is a balance between the need to preserve key populations of rare plants and limitation of intervention. However, this balance depends on how intervention is defined. The introduction of A. grandiflora is seen by its opponents as artificial because plants resulting from expected crosses between plants from Fontainebleau and Chinon are a product of human action. In the same way, one naturalist criticized the creation of new sub-populations at locations where the species had never been observed. Such actions are criticized because they alter what is seen as the nature of nature: its spontaneity, its autonomous force, independent of human will. This critique can be seen as a reaction against a phenomenon that previous social studies underlined: the emergence of new categories of wild nature. These new categories are tightly linked with practices of ecological engineering (introduction of species, modification of environmental conditions to favor one species) and have been described primarily based on animal studies [44],[45],[46]. These views are incompatible with the traditional conception of wild nature based on an opposition to human intervention: nature is what is outside of the domestic space, what has not been directly modified by human action. The emergence of these new categories of wild nature can be observed in the same way with plants. In the case of the naturalists opposed to the introduction of A. grandiflora, there is conflict between these new categories of nature and the naturalists' conception of nature, which is usually close to the traditional one.

In parallel, instigators of the introduction recognized that they practice proactive management, which to some looks like “nature gardening”, and acknowledged that the use of plants from another isolated population is not trivial. However, they felt that this program was the last chance and the result of the failure of all other solutions. They considered the active management of *A. grandiflora* populations necessary for eventually achieving self-sustainability. As a result, they were willing to consider a new category of wild nature. Moreover, they tempered their actions by claiming that their goal was not to “recreate nature” but to facilitate the population's recovery with a one-time intervention. The population would be left to evolve naturally in the future. This highlights another way in which the views of the various actors of this conservation plan are opposed, this time based on temporal consideration of their actions. Indeed, the advocates of the introduction focused on the present and the future simultaneously; their action helps to maintain the future population but remains brief on the scale of evolutionary mechanisms. By contrast, naturalist opponents analyzed the plan by referring to the past, i.e., the botanical heritage that the plant represents. Their personal experience is based on floras, herbaria, and more or less recent *in situ* observations, witnesses of the past of the plant.

The interviews highlighted that the limit of the intervention was a major point of disagreement. Some words used by naturalists and scientists to qualify the restoration plan belong to the lexicon of medicine and ethics: “gene therapy”, species “under perfusion”, “therapeutic relentlessness”. This language underlines the controversy regarding the use of recently developed sophisticated techniques on living organisms and is employed to describe the program as an attempt to maintain populations whatever their state, in other words, as an action without appropriate ethical consideration. In the same way, a naturalist compared the reintroduction to the use of genetically modified organisms. This example represents an illustration of a myth about the research world, depicting it as an independent entity that society should be protected from [47]. This myth, occurring outside of the scientific world or, here, outside of a specific discipline (e.g., population genetics), would operate to protect society from the dangers of objective science, of technologies, and of rationalization [47].

## Conclusion

The extent of the experiment, the intensity of its survey, and its coupling with a socio-anthropological study mark the restoration plan for *Arenaria grandiflora* L. in the Fontainebleau forest as one of the first experiments of its kind. This work provided an opportunity to study the introduction of non-local individuals to counteract inbreeding depression among local individuals to establish the benefit of mixed populations in cases such as this one. The results indicate that the choice of suitable restoration sites may be one of the main factors affecting the success of plant introductions. This case study suggests that the ethical position of different participants involved in restoration programs may vary with their knowledge (i.e., not only of biogeography, ecology, or evolution but also of botanical history, personal observations of landscape evolution, and population biology concepts), and therefore introduction plans may result in controversy. Hence, the elaboration of restoration programs for threatened populations should consider both ecological concepts and their social meanings for the different protagonists. The knowledge of local people interested in the restoration plan should be taken into account to facilitate mutual comprehension. Sociological studies through early interviews of the different protagonists could help identify points of disagreement and avoid counterproductive conflicts.
